# Relatedness of White-Tailed Deer from Culling Efforts Within Chronic Wasting Disease Management Zones in Minnesota

**DOI:** 10.3390/pathogens14010067

**Published:** 2025-01-13

**Authors:** Alberto Fameli, Christopher Jennelle, Jessie Edson, Erik Hildebrand, Michelle Carstensen, W. David Walter

**Affiliations:** 1 Pennsylvania Cooperative Fish and Wildlife Research Unit, The Pennsylvania State University, University Park, PA 16802, USA; 2 Nongame Wildlife Program, Minnesota Department of Natural Resources, 500 Lafayette Road, St. Paul, MN 55155, USA; 3 Wildlife Health Program, Minnesota Department of Natural Resources, 5463 West Broadway Ave., Forest Lake, MN 55025, USA; 4 U.S. Geological Survey, Pennsylvania Cooperative Fish and Wildlife Research Unit, 403 Forest Resources Building, The Pennsylvania State University, University Park, PA 16802, USA

**Keywords:** chronic wasting disease, culling, Minnesota, *Odocoileus virginianus*, white-tailed deer, population genetic structure, relatedness, wildlife disease management

## Abstract

In white-tailed deer (*Odocoileus virginianus*), closely related females form social groups, avoiding other social groups. Consequently, females infected with chronic wasting disease (CWD) are more likely to infect social group members. Culling has been used to reduce CWD transmission in high-risk areas; however, its effectiveness in removing related individuals has not been assessed. We analyzed 11 microsatellites and a mitochondrial DNA fragment to assess: (1) the genetic structure in white-tailed deer in Minnesota, USA and (2) the effectiveness of localized culling to remove related deer. For (1), we genotyped deer culled in 2019 and 2021 in three CWD management zones, and deer collected in between zones. For (2), we only included culled deer, defining “culled groups” as deer obtained in the same township-range-section and year. We compared mean relatedness among deer from the same culled group (intra-group relatedness) and among deer from different culled groups (inter-group relatedness). We did not find evidence of genetic structure, suggesting that an outbreak in any of the management zones could naturally spread to the others. Culling removed deer that were on average more related than expected by chance (intra-group relatedness > inter-group relatedness), and most highly-related deer were culled in the same bait site.

## 1. Introduction

Chronic wasting disease (CWD) is a transmissible spongiform encephalopathy affecting cervids [1,2]. As is the case with all prion diseases, CWD is characterized by an invariably fatal neurodegenerative process; however, CWD stands out for being the only prion disease affecting free-ranging species [3,4], and is rapidly expanding in geographic range [5]. Some studies have identified CWD as a significant contributor to cervid population declines, reducing survival rates to levels incompatible with long-term persistence [6,7,8]. Infected individuals shed prions in saliva, feces, antler velvet, and urine [9,10,11,12], and transmission occurs through contact with infected individuals or via the ingestion of prions persisting in the environment [13,14,15].

Given the lack of treatment or a vaccine, management plans focus on population reduction through increased recreational hunting and/or culling by government agencies [16,17,18,19], which assumes density-dependent transmission of CWD [20,21,22]. For wild white-tailed deer (*Odocoileus virginianus*), strictly density-dependent transmission is unlikely given host behavior, as female philopatry creates social groups of closely related females and their fawns, with reduced contact between social groups [23,24,25]. Males have a higher risk of CWD infection, which could be explained by their larger home ranges increasing their probability of coming in contact with CWD [26]. By contacting multiple females during breeding season, males could also act as drivers of CWD expansion while philopatric groups of females maintain local infectious foci.

While population density may play a role in some cases, for example during congregations around baiting sites, mineral licks, and winter ranges [22,27,28], studies have shown that CWD transmission in white-tailed deer is mainly frequency-dependent, with infectious contact most likely occurring among closely related individuals from the same social group, even when multiple social groups overlap in space [29,30,31]. As a result, kinship between individuals can serve as a more accurate indicator of the contact rate than spatial location due to avoidance between neighbors from different social groups [32]. For example, Grear et al. conducted a study in an area of advanced-stage CWD (prevalence ~6.5%) and found that the probability of infection in adult females increased over 100-fold with the presence of a closely related, CWD-positive female located <3.2 km away [33]. The presence of less-related CWD-positive females at the same distance had a much smaller effect on the probability of infection. While the specific value of a 100-fold increase in infection probability depends, among other factors, on the prevalence of CWD in the area, the study still unveils an important demographic scenario in which deer from the same area can differ substantially in their probability of infection, as direct transmission among females and juveniles seems mostly restricted to social groups, and genetic relatedness can be a determinant for the probability of infection. This scenario encourages a shift in the target of population reduction for CWD management from individuals (generalized public hunting) to social groups, by attempting to remove as many individuals of a social group as possible.

Localized culling may be more effective than generalized public hunting in removing social groups, thus lowering within-group contacts, which in turn would lower CWD spread and transmission. Localized culling prevented CWD establishment in New York and Minnesota, United States and Ontario, Canada [34,35,36], while severely depressing its prevalence in Illinois, United States [17]. Although in Minnesota, United States, CWD was detected for the first time in free-ranging deer in 2010, near an infected captive elk herd, CWD appeared to have been eliminated until 2016, when CWD was again detected in southeastern Minnesota in several free-ranging deer and currently persists at low levels in that area. In response, the Minnesota Department of Natural Resources (MNDNR) implemented an ambitious surveillance and management program [37], including increased harvesting, culling, feeding bans, and carcass movement restrictions. Targeted agency culling was first utilized by the MNDNR during an outbreak of bovine tuberculosis in the mid-2000s [38]. Culling in response to CWD was implemented in 2011, 2017, and four consecutive years (2019, 2020, 2021, and 2022) in winter.

During winter, deer from different social groups may visit the same feeding areas, albeit not moving together as a herd [23], a behavior that could be exacerbated by the use of baiting in culling operations [39]. Furthermore, not all deer from the same social group approach bait sites together [40] or are culled simultaneously, which results in incomplete removal of the social group during a single culling event. Surviving individuals may or may not return to the bait site. A combination of these phenomena makes it unclear whether deer culled in a site asynchronously over days/weeks encompass social groups of related individuals or rather represent a collection of unrelated deer from different social groups. Rosenberry et al. found a lack of mother–offspring pairs in groups of co-captured deer in central Pennsylvania, USA, possibly a result of pre-capture harvesting and incomplete representation of social groups in captures [40]. On the other hand, Robinson et al. in Wisconsin, USA applied a more targeted capture strategy than the previous study, obtaining higher success in co-capturing closely related individuals [41].

Kinship between wild individuals is difficult to estimate accurately due to factors such as incomplete sampling (i.e., one or both parents missing), overlapping generations, and a lack of information on age [42]. Incorporating information such as sex and mitochondrial haplotype can improve kinship estimates [42,43]. Another option is the use of methods that do not attempt to classify individuals into discrete classes (e.g., parent-offspring), but instead calculate relatedness: a continuous measure representing the likelihood that alleles shared by individuals are identical by descent [42,43]. The objectives of a study determine the level of accuracy needed; relatively few loci can be sufficient when the goal is to compare groups of samples based on their average relatedness [43].

Population genetic structure (i.e., the presence of demes with different allelic frequencies) needs to be evaluated prior to assessing kinship, because estimates of allelic frequencies are necessary to calculate relatedness/relationships [44,45,46]. Assessing the host genetic structure also has intrinsic value, allowing to infer connectivity, which can play a significant role in the natural spread of CWD, but which has not been evaluated for white-tailed deer in Minnesota.

Our first objective was to evaluate the possibility of spatial or temporal population genetic structure across a wide north–south gradient in Minnesota, which would preclude us from comparing deer of different zones or periods when estimating relatedness/relationships. Our second objective was to evaluate the potential of culling operations implemented by the MNDNR to remove related individuals from first-priority areas for culling, which could represent the removal of social groups. To this end, we compared the mean relatedness of deer culled in the same area over days/weeks (i.e., “culled group”) vs deer culled in different areas. We expected to find no difference between our two means of relatedness if the culled groups were mostly comprised of individuals from different social groups. Conversely, a higher mean relatedness among deer from the same culled group may indicate the effectiveness of culling operations in removing multiple deer belonging to the same social groups, a result for CWD management. We also evaluated the presence of mother–offspring pairs and full siblings among our culled deer, which are presumed to have belonged to the same social group based on known deer behavior. We did not find evidence of genetic structure, suggesting that an outbreak in any of the management zones could spread to others by natural deer movements. Culling operations removed deer more related than expected by chance, and most pairs of full siblings and mother–offspring detected were culled in the same site, even when culled weeks apart. This indicates that localized culling can weaken social groups, potentially decreasing disease prevalence. To our knowledge, we are the first to evaluate the genetic relatedness/relationships among deer removed from a wild population during culling operations.

## 2. Materials and Methods

**Figure 1:**
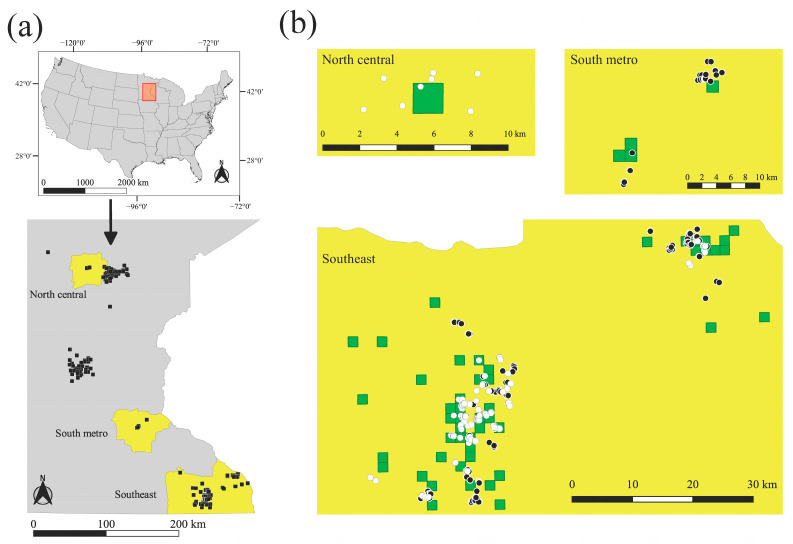
Distribution of white-tailed deer (*Odocoileus virginianus*) samples from Minnesota, USA. (**a**) Samples included to better represent allelic frequencies in the population (i.e., hunter-harvested, killed in vehicle collisions, found dead, or selectively removed during other operations; *n*_other_ = 203) collected between 2016–2021, represented by black squares. Current disease management zones are represented in yellow: North central, South metro, and Southeast. (**b**) Samples from deer culled in disease management zones in 2019 (white circles, *n*_2019_ = 511) and in 2021 (black circles, *n*_2021_ = 542). Green squares are 2.6 km^2^ and represent township-range-sections where chronic wasting disease was detected in previous years (i.e., first-priority areas).

We conducted our study in an area of about 43,500 km^2^ in central and southeast Minnesota, USA, although sampling was concentrated mainly in three CWD management zones: southeast, south metro, and north central (Figure 1a). Deer density within the permit areas belonging to the management zones was estimated to be between 19 and 41 deer/mi^2^ ≈ 2.6 km^2^ for the years included in our study [47]. The MNDNR delineated first-priority areas for deer culling efforts; each one equals the township-range-section (quadrant of 1 mi^2^, approx. 2.6 km^2^, as defined by the USA Public Land Survey System) where a CWD-positive deer was previously detected. We included deer from two culling seasons, January–March of 2019 and February–March of 2021 (Figure 1b). There were 56 first-priority areas in our study; however, access to deer was limited by private landowner permission and the characteristics of the terrain, in which case the MNDNR conducted culling in surrounding areas when possible. This resulted in 30 first-priority areas where culling took place: 17 in 2019 and 13 in 2021. On average, 14 deer (min = 3, max = 37) were culled in each first-priority area. A muscle sample was collected from each individual for genetic analysis (*n*_2019_ = 511; *n*_2021_ = 542; [48]).

We included additional samples (*n*_other_ = 203, Figure 1a) to better represent the population in the area, collected between 2016–2021 from hunter-harvested deer, deer killed in vehicle collisions, selectively removed by the MNDNR during other operations, or found dead. Some of these samples lacked geographic coordinates; therefore, we assigned them to the centroid of their township-range-section of origin. We stored samples at −20 °C, then cut a slice of approximately 1 × 3 × 5 mm from each sample, placed them on card stock to dry for 24–48 h at approximately 19 °C, and stored them at room temperature until DNA extraction.

We extracted DNA using Dneasy blood and tissue kit (Qiagen Inc., Valencia, CA, USA). Following Miller et al. [49], we amplified a panel of 11 microsatellites: INRA011 [50], OarFCB193 [51], BL42, BM6438, BM4107 [52], Cervid1 [53], RT7, RT5, RT9 [54], OvirP, and OvirQ [55]. Alleles were called independently by two researchers, either using GENEIOUS v6.0.6 [56] or a combination of GENEMARKER v2.6.3 (SoftGenetics LLC, State College, PA, USA) and the MSATALLELE package [57] in R v4.0.0 [58]. In case of differences, we reached a consensus by additional genotyping.

We sequenced a fragment of about 546 bp belonging to the mitochondrial (mtDNA) control region using both forward and reverse primers [59]. We used GENEIOUS v6.0.6 to inspect the quality of sequences and trim them to a 471 bp fragment, keeping only those that had at least 75% of their bases with a Phred quality score of 20 (i.e., 99% accuracy in base calling). We used MEGA-X v10.2.5 [60] to produce sequence alignments via the MUSCLE algorithm [61], and obtained a consensus based on the forward and reverse sequences. We used ARLEQUIN v3.5.2.2 [62,63] to group samples according to their haplotype.

### 2.1. Population-Level Genetic Analyses

#### 2.1.1. Evaluation of Marker Panel

This study included analyses meant to describe the deer population in the study area and individual-based analyses. These two scales required analysis using different sets of samples, because our clumped sampling could result in overestimating the number of population clusters [64,65] or biasing the linkage-disequilibrium test [66]. Using one sample per township-range-section (*n*_subset_ = 200), we ran MICRO-CHECKER v2.2.3 [67] to assess the possibility of genotyping errors due to null alleles, large allele dropout, or stuttering, using a Bonferroni-adjusted 95% confidence interval. We also tested our markers for non-random association of alleles at different loci, using the linkage disequilibrium test implemented in ARLEQUIN v3.5.2.2 with 10,000 permutations (Bonferroni-corrected α = 0.001).

#### 2.1.2. Temporal Genetic Structure

We assessed the possibility of temporal differences in allelic frequencies, which would preclude us from pooling together samples from different years for the subsequent analyses. We focused on the southeast management zone as it was the only one with enough samples in multiple years, and assumed this zone was representative of the study area. For each year, we used one sample per township-range-section in this management zone (*n*_2017_ = 12; *n*_2018_ = 12; *n*_2019_ = 62; *n*_2020_ = 21; *n*_2021_ = 48). Using ARLEQUIN v3.5.2.2, we compared years regarding the number of different alleles (F_ST_, as carried out by Fedy et al. [68]) and the sum of the squared allele size differences (R_ST_, as carried out by Winton et al. [69]) with 10,000 permutations to assign significance (Bonferroni-corrected α = 0.005).

#### 2.1.3. Global Genetic Structure

We used two methods to evaluate the possibility of spatial population genetic structure, including only one sample per township-range-section (*n*_subset_ = 200). First, we ran a spatial principal component analysis (sPCA, [70]), a multivariate method explicitly including geographic locations to allow the detection of genetic patterns, using ADEGENET package v2.1.3 [71] in R v4.0.0. Principal components in sPCA are composite: they maximize variance (i.e., genetic diversity) and spatial autocorrelation [72]. These principal components can show positive autocorrelation, representing “global” patterns (i.e., neighbors are more similar than expected by chance, as in the case of clines or clusters) or negative autocorrelation, representing “local” patterns (i.e., neighbors are more different than expected by chance, [70]). We based our analysis on a Delaunay triangulation network to define neighbors, and tested for global and local patterns using the SPCA_RANDTEST function [72] (α = 0.05) with 9999 permutations. We used the SCREEPLOT function to create a graph showing the decomposition of eigenvalues and to choose which principal components to interpret [73].

The second method we used was the Bayesian clustering approach, implemented by STRUCTURE v2.3.4 [74] with the following settings: burn-in period = 500,000 steps followed by 1,000,000 steps, number of assumed clusters (K) = 1–10, and 10 runs per K. We used an admixture model and correlated allele frequencies [64], leaving the remaining parameters on their default values. We evaluated the results with STRUCTURE HARVESTER web v0.6.94 [75] to select the best-supported number of clusters, based on the mean ln(likelihood) and Evanno’s ΔK [76]. We used CLUMPAK [77] to check for convergence of the runs and to create average bar plots representing the membership of each deer to the assumed clusters.

#### 2.1.4. Fine-Scale Genetic Structure

To examine the possibility of sex-specific spatial structure at a fine scale, we carried out a spatial autocorrelation analysis [78] using the test implemented in GENALEX v6.5 [79,80]. This analysis partitions the geographic distance between individuals into discrete classes, and inside each class it calculates the autocorrelation coefficient between individuals based on their genetic and geographic distance. We conducted this test for all adult males (*n* = 228) and adult females (*n* = 529) separately, using 20 distance classes of 1 km. We did not include yearlings and fawns, as we wanted to represent the structure after natal dispersal [81]. Due to their philopatry, we expected females to be more genetically similar than males at short geographic distances. We applied both methods available in GENALEX to test for significance: random permutation (999 permutations) and bootstrap (999 bootstrap trials), in both cases assigning significance based on a 95% confidence interval. Because some samples originally lacked collection coordinates and were assigned to the centroid of their township-range-section of origin, they ended up having a geographic distance of zero, despite being potentially separated by up to 2.28 km. This was the case for only two females and two males. We repeated the spatial autocorrelation analysis, removing all samples that lacked collection coordinates (5.9% of adult females and 11.8% of adult males).

### 2.2. Section-Level Genetic Analyses

#### Relationships and Relatedness Among Culled Individuals

We evaluated the presence of full siblings (FS) and mother–offspring (MO) pairs among culled deer using COLONY v2.0.6.8 [82], which searches for the best relationship assignments based on the maximum likelihood and allows users to incorporate additional information, improving assignments. We did not assess the presence of half-siblings because they do not necessarily share an mtDNA haplotype, preventing us from using this information to exclude spurious assignments. We performed this analysis for all individuals culled in 2019 and separately for individuals culled in 2021, considering fawns and yearlings as offspring, adult males as potential fathers, and adult females as potential mothers. Yearling females were included as potential mothers only for fawns [83]. For each offspring, we excluded as potential mothers all females whose mtDNA haplotype did not match that of the offspring in question. We excluded as FS all other offspring that did not share an mtDNA haplotype with the offspring in question. Due to the relatively few biparental markers used, we decided not to infer relationships among adult deer since we could not make use of age to assist in the assignment. We used the following settings: male and female polygamy [84], no inbreeding (population is assumed to be in Hardy–Weinberg equilibrium), no clones, long runs, full-likelihood method (the most accurate one according to [85]), medium likelihood precision, no updating of allele frequency to account for inferred relationships, no sibship scaling, and no sibship size prior. We tested different average probabilities of the actual mother/father of offspring being included in the sample. For each year, we conducted four separate runs with different combinations of these parameters: probability of including mother = 0.5 or 0.7, probability of including father = 0.01 or 0.2. We decided to interpret only FS and MO pairs inferred at a probability >0.8 in at least three out of the four runs for each year. We computed the geographic distance between FS and MO using QGIS v3.8 [86].

We calculated a moment estimator of pairwise relatedness (*r_QG_*, [87]) between individuals using COANCESTRY v1.0.1.9 [88]. In cases like ours, where allele frequencies are estimated from the sample itself instead of a reference population, *r_QG_* is better interpreted as a correlation coefficient of homologous alleles between individuals [89]. Therefore, the *r_QG_* for a dyad of individuals can take values between −1 and 1, with negative values indicating lower-than-average relatedness and positive values indicating higher-than-average relatedness [90]. We classified deer into culled groups: those culled in the same township-range-section and year were assigned to the same culled group. We only included samples from first-priority areas. This resulted in 17 culled groups for 2019 (3–37 deer per group, average = 17) and 13 culled groups for 2021 (3–26 deer per group, average = 11). We used the group difference test included in COANCESTRY to evaluate if the mean *r_QG_* between deer from the same culled group (i.e., intra-group *r_QG_*) was different than the mean *r_QG_* between deer from different culled groups (i.e., inter-group *r_QG_*). In this test, dyads of individuals are classified into two categories and the mean *r_QG_* is calculated for each category. The difference between these two observed means is compared to the expected difference of means, obtained by bootstrapping. We used 10,000 bootstrap samples to build a 95% confidence interval, considering the observed difference of means as statistically significant if it fell outside the defined interval [91]. We performed this intra-group vs inter-group comparison separately for deer culled in 2019 and culled in 2021, plotting the results with DPLOT Jr v2.2.2.1 (HydeSoft Computing LLC, Vicksburg, MS, USA). Because high intra-group relatedness could be ascribed solely to the presence of mother–fawn pairs, we repeated the analysis excluding fawns. We also repeated this analysis using samples only from the southeast management zone, to avoid comparing individuals from very distant areas in our estimation of mean inter-group relatedness.

## 3. Results

### 3.1. Population-Level Genetic Analyses

We identified the presence of null alleles at loci BL42 and OvirQ. The latter also showed a pattern of allele sizes inconsistent with its reported motif, as described by Miller et al. for samples collected in northeast USA [49], with some alleles separated from one another by only 1 bp. Following a conservative approach, we decided to exclude OvirQ from the analyses. We found no evidence of linkage disequilibrium between the remaining loci (*p*-values ≥ 0.008, Bonferroni corrected α = 0.001). The number of alleles per locus ranged between 9 and 16 (mean = 13.6). We obtained at least one reliable mtDNA control region sequence for 1249 of 1256 samples, representing 58 haplotypes.

Pairwise comparisons between years in the southeast management zone showed no evidence of differences in allelic frequencies, both for F_ST_-based and R_ST_-based comparisons (*p*-values ≥ 0.059 and ≥0.130, respectively, Bonferroni-corrected α = 0.005). As a result, we pooled together samples from different years.

**Figure 2:**
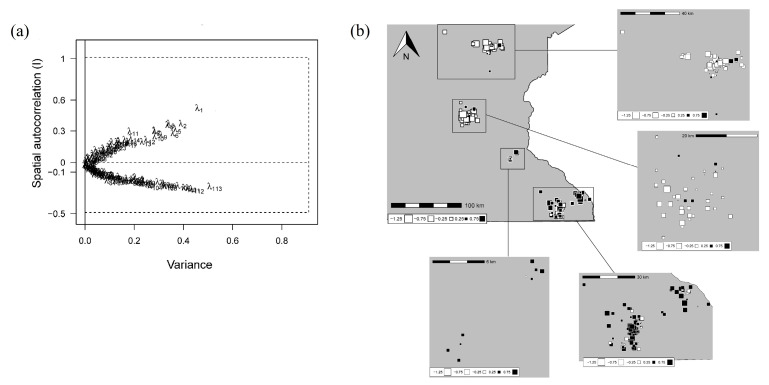
Results of spatial principal component analysis based on a subset of white-tailed deer (*Odocoileus virginianus*) genetic samples (i.e., one sample per township-range-section, *n*_subset_ = 200) used to evaluate the presence of a geographical genetic structure in Minnesota, USA. Samples were collected between 2016 and 2021. (**a**) Decomposition of eigenvalues into their variance and spatial autocorrelation. We decided to interpret the first (λ_1_) principal component. (**b**) Samples represented by squares, with their size and color indicating their value on the first principal component.

**Figure 3:**
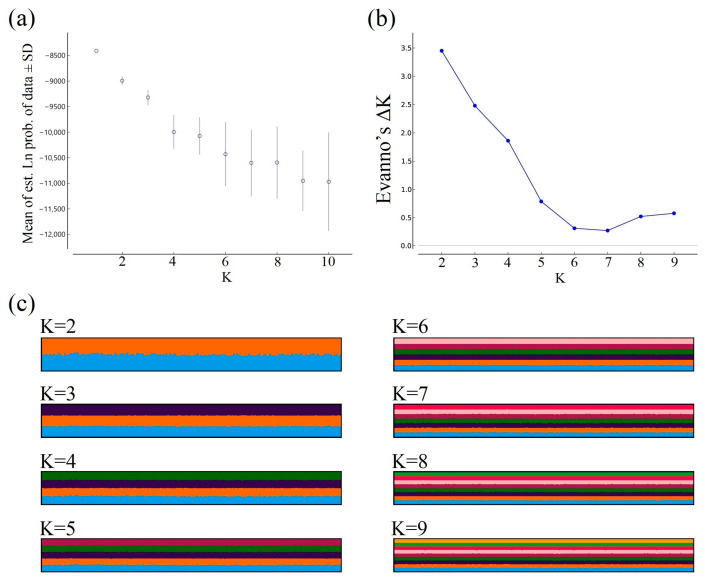
STRUCTURE results based on a subset of white-tailed deer (*Odocoileus virginianus*) samples (i.e., one sample per township-range-section, *n*_subset_ = 200) used to evaluate the presence of a geographical genetic structure in Minnesota, USA. Samples were collected between 2016 and 2021, and we tested the hypotheses of 1 to 10 clusters present (K), with 10 independent runs per K. (**a**) Mean logarithm of estimated posterior probability ± standard deviation (SD) vs. K. The highest mean logarithm of the estimated posterior probability was obtained for K = 1, consistent with a scenario of no genetic structure. (**b**) Evanno’s ΔK vs. number of clusters. This statistic is based on the difference in the mean logarithm of the estimated posterior probability between successive K values. The highest ΔK was obtained for the difference between K = 1 and K = 2, which could indicate the presence of one or two clusters. (**c**) Barplots showing membership of individuals to different assumed clusters. Each vertical bar represents an individual and the colors show membership to each of the assumed clusters. In a scenario where there are multiple genetic clusters present, it would be expected that some individuals are represented mostly by one color, showing their membership to one of the clusters. Note that in our case, all individuals had equal membership to all genetic clusters being considered, which is consistent with absence of a genetic structure (i.e., K = 1).

We interpreted the first principal component of the sPCA based on eigenvalue decomposition (Figure 2a); however, this axis only explained <1% of the total variance. The map displaying the spatial distribution of samples and their value on this axis did not show a clear pattern of structure (Figure 2b), as samples with positive and negative values were found across our study area. This was confirmed by the test of global and local spatial patterns, which showed no support to the presence of either pattern (*p*-values 0.066 and 0.072, respectively). The STRUCTURE results showed that the mean ln(likelihood) was highest for K = 1 and decreased as K increased (Figure 3a), suggesting the presence of only one genetic cluster in our study area. Evanno’s ΔK also decreased as the number of assumed clusters increased, except for K = 8 and K = 9 (Figure 3b). Membership barplots were consistent with K = 1, showing approximately equal partitioning of each individual’s genome into the assumed clusters (Figure 3c). All runs for each K value converged to the same clustering solution except for K = 2 (9/10 runs converged to the result shown in Figure 3c) and K = 3 (6/10 runs converged to the result shown in Figure 3c), but differences between solutions were minimal and did not affect interpretation of the underlying pattern.

**Figure 4:**
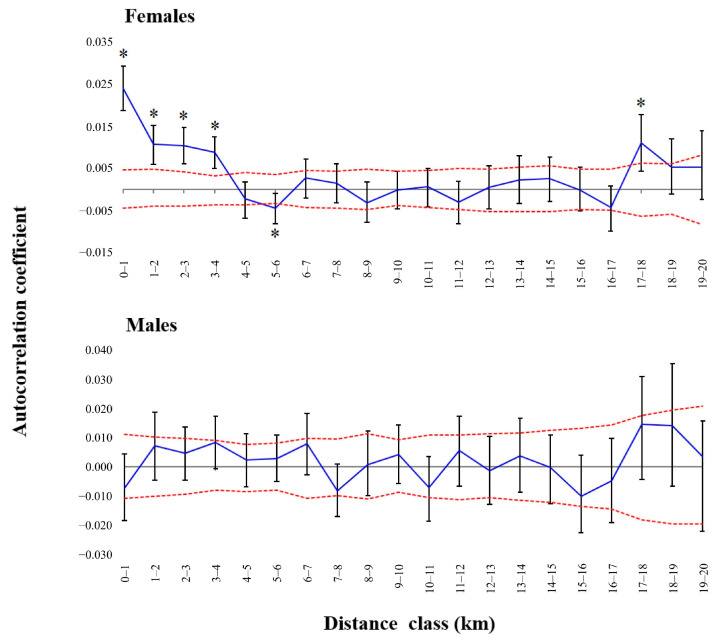
Results of the spatial autocorrelation analysis between genetic and geographic distance for adult female and male white-tailed deer (*Odocoileus virginianus*) in Minnesota, USA. Samples were collected between 2016 and 2021. Dashed lines represent upper and lower bounds for the 95% confidence interval under the assumption of no spatial structure, obtained through random permutation. Error bars around each observed autocorrelation coefficient represent the 95% confidence interval obtained by bootstrapping. Statistically significant values (i.e., those outside the area delimited by dashed lines and those whose error bar does not straddle zero) are signaled by an asterisk.

Adult females displayed a pattern of positive autocorrelation up to a distance of 4 km, being more genetically similar than expected by chance, which was significant for both statistical methods implemented (Figure 4). We also found significant negative correlation for females between 5 and 6 km apart, and positive autocorrelation at 17–18 km. Conversely, adult males did not show significant autocorrelation for any of the distance classes evaluated. Even after removing all samples lacking coordinates and repeating the analysis, we obtained the same pattern of positive autocorrelation for females up to a distance of 4 km and no autocorrelation for males.

### 3.2. Section-Level Genetic Analyses

For deer culled in 2019, we inferred 15 pairs of FS (Table 1) with assignment probabilities of between 0.825 and 1. All these pairs were separated by <400 m, even when individuals were culled several days apart. For the same year, we detected 73 MO pairs: 56 adult-fawn, one yearling-fawn, and 16 adult-yearling. Most of these MO pairs (82%) were culled on the same bait site or separated by <1 km, even when they were culled weeks apart. A female fawn was culled >11 km from its inferred mother, 30 days later.

For individuals culled in 2021, we inferred 12 pairs of FS, although one of them was clearly incorrect, as it was between two fawns of different mtDNA haplotypes. Most of these FS pairs (82%) were culled on the same bait site, even when individuals were culled weeks apart. The remaining FS pairs were separated by more than 50 km, but these were fawn–yearling pairs, where the latter could represent a disperser. For the same year, we inferred 67 MO pairs: 51 adult–fawn, 1 yearling–fawn, and 15 adult–yearling. Again, most of these pairs (91%) were culled on the same bait site or separated by <1 km, even when they were culled weeks apart. According to these results, an adult female was culled 144 km from her fawn, 22 days later.

**Figure 5:**
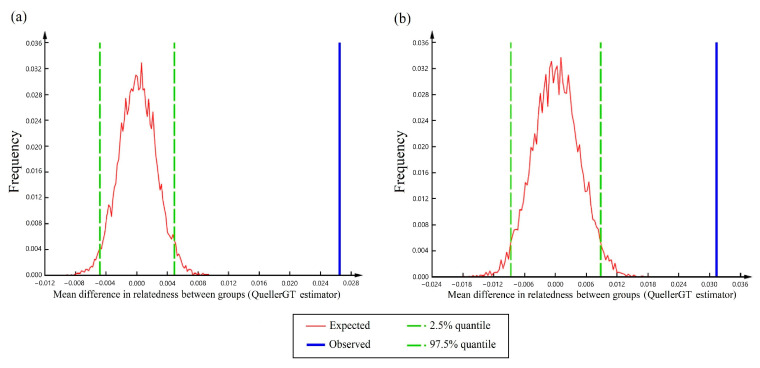
Difference in mean relatedness between white-tailed deer (*Odocoileus virginianus*) from the same culled group vs deer from different culled groups, for samples obtained during (**a**) 2019 and (**b**) 2021 in Minnesota, USA. We calculated relatedness using the moment estimator QuellerGT proposed by Queller and Goodnight (1989) [87]. The blue line represents the observed difference in mean relatedness between deer belonging to the same culled group vs. deer from different culled groups, while the red line represents the difference between those categories obtained by bootstrapping (i.e., expected difference of means) and its frequency. The boundaries of the 95% confidence interval for the expected difference of means are shown in green.

From the 30 culled groups we defined, 27 contained females and fawns representing multiple mtDNA haplotypes, suggesting that most culled groups contained more than one social group. For 2019, the relatedness estimated between deer from the same culled group took values of between −0.3 and 0.7, while relatedness between deer from different culled groups took values of between −0.3 and 0.4. For 2021, the relatedness estimated between deer from the same culled group took values of between −0.3 and 0.7, while the relatedness between deer from different culled groups took values of between −0.4 and 0.4. The relatedness analysis revealed that the mean intra-group *r_QG_* was significantly higher than the mean inter-group *r_QG_* for both culling years (Figure 5), indicating that pairs of deer culled in the same township-range-section were on average more related than other pairs. In 2019, the difference between the mean intra- and inter-group *r_QG_* was 0.026, outside the expected difference 95%CI = [−0.005; 0.005] (Figure 5a). In 2021, the difference between the mean intra- and inter-group *r_QG_* was again significant (observed difference = 0.031, expected difference 95%CI = [−0.009; 0.009], Figure 5b). These results were maintained after the removal of fawns from the analysis: for 2019, the observed difference of means was 0.022 and the expected 95%CI = [−0.007; 0.007], while for 2021, the observed difference was 0.028 and the expected 95%CI = [−0.012; 0.012].

Restricting our analysis to the southeast management zone produced the same results: for 2019, the observed difference of means was 0.026, outside the expected 95%CI = [−0.005; 0.005], while for 2021, the observed difference was 0.027, outside the expected 95%CI = [−0.009; 0.009]. 

## 4. Discussion

The lack of genetic structure found in white-tailed deer for our study area could be due to gene flow at rates that prevent genetic differentiation, or the presence of barriers to gene flow that are too recent to be detected. Our results likely represent the former, because a high dispersal capacity has been documented for white-tailed deer in Minnesota, at a maximum distance of 168 km [24] and 117 km [92]. A lack of genetic structure was also reported across an extensive area covering southern Wisconsin and northern Illinois [93], and between northeast Iowa and Wisconsin, where researchers found connectivity even across the Mississippi River [94]. Some studies did find a genetic structure in deer using the same methods we applied, such as Hopken et al. near the border between Oregon and Washington, USA [95]. This indicates that a lack of genetic structure cannot be assumed on the basis of the species’ high dispersal capacity. We conclude that the white-tailed deer in our study represent a single demographic unit, suggesting that in the absence of effective management actions, an outbreak in any of the management zones could potentially spread to the others by natural deer movements.

Spatial autocorrelation analysis showed differences in the fine-scale population genetic structure between adult males and females. We detected significant autocorrelation in adult females at a distance of 17–18 km, showing that some females are able to disperse to at least this distance, a result corroborated by deer equipped with global positioning system collars [92]. Our results suggest a higher philopatric behavior in females, a pattern we expected based on our knowledge of the species, but whose extent had not been evaluated previously in Minnesota. The extent of continuous spatial autocorrelation for adult females (4 km) was larger than reported in other studies. Miller et al. found positive autocorrelation up to a distance of ≈1 km in West Virginia, USA [96], while Cullingham et al. found it to be <1 km in an area spanning from British Columbia to Saskatchewan, Canada [97]. Conversely, weak correlation was found between genetic and geographic distances in female deer from South Carolina, USA [81], possibly a result of high adult mortality associated with intense harvesting preventing the establishment of a typical “rose-petal” social structure [98] and increasing the dispersal of orphaned female fawns. Different degrees of social structuring displayed by separate populations indicate that the effectiveness of (and need for) localized culling to remove related deer should be considered on a population-specific basis.

The pattern of genetic spatial autocorrelation we found does not guarantee by itself the effectiveness of culling to remove closely related individuals. We found that numerous closely related deer (FS and MO) were culled on different days, suggesting that a single culling session in a location would often fail to remove all deer from a social group. Furthermore, culling operations in Minnesota could have created shifts in deer space use, as reported by researchers in Illinois who found that remnant juveniles after culling showed lower site fidelity [99]. These researchers concluded that such disruption in remnant juveniles could favor disease spread, and suggested that disease management actions should remove entire social groups. While we did not analyze shifts in space use, we reported spatial and temporal distances between inferred FS and MO pairs and found that most individuals from the same pair were culled <1 km from each other even when culled weeks apart, suggesting that most individuals stay in or quickly return to their original home range.

Determining the effectiveness of culling as a CWD management tool is challenging due to all the variables that contribute to potential disease spread combined with multiple control strategies being implemented simultaneously. The apparent prevalence of CWD in southeastern Minnesota has remained at approximately 1% over the past 5 years; however, evaluating the contribution of culling to lower prevalence versus increased hunting pressure, prohibition of recreational feeding and use of attractants, or carcass disposal programs to reduce the environmental burden of prions remains difficult. As a first step to evaluate the white-tailed deer culling plan implemented in Minnesota, we assessed its effectiveness in removing individuals that were more related than average. We found that deer culled in the same first-priority area were on average more related than deer culled in different first-priority areas. This result is not trivial; deer culled in the same area could represent a collection of individuals from different social groups or include a high proportion of adult males, which are assumed to be transient and have dispersed from other areas. In fact, most culled groups we defined (27 out of 30) included females/fawns representing different mtDNA haplotypes, indicating the presence of multiple matrilines in each first-priority area and confirming the spatial overlap of different social groups. Nonetheless, the mean relatedness between deer from the same culled group was higher than between deer from different culled groups. This finding can be explained by the culled groups containing multiple related deer, which increased the value of the average intra-group relatedness despite the presence of multiple matrilines. We attribute these results to the general effectiveness of culling operations in removing multiple individuals from the same social groups. These results were not ascribed to the presence of mother–fawn pairs inflating the mean relatedness between deer from the same culled group, as we found the same pattern after removing fawns from the analysis. Even when we restricted our analysis to the southeast CWD management zone, we found that the mean relatedness was higher between deer in the same culled group.

Whether culling operations in Minnesota removed all members from each social group is unknown, but our results documented that localized culling by the MNDNR was capable of removing multiple deer that were more related than expected by chance in each first-priority area, despite also removing individuals from different social groups. Consequently, these operations have the potential to eliminate infectious foci, besides their more obvious effect in reducing local population density. Future research using more genetic markers could provide additional details on parentage within culled groups to further evaluate the efficacy of culling operations to achieve project objectives. On average, 14 deer were culled in each first-priority area (2.6 km^2^), while hunter-harvesting in disease management areas (where there is no limit on antlerless harvest and there is an early antlerless season) removed on average 4.9 deer/2.6 km^2^ and 6.4 deer/2.6 km^2^ in 2019 and 2021, respectively [100,101]. The higher localized intensity in deer removal through culling, compared to harvesting, even when the latter has reduced restrictions, could mean that culling removes more individuals from the same social groups than harvesting. A comparison of relatedness among the deer removed through hunter-harvesting, designed similar to our culling study design, could provide insight into the effectiveness or limitations of using hunting alone as a disease control strategy when culling is not considered a viable option.

Our evaluation of culling operations carried out in Minnesota indicates that persistent culling in a first-priority area can achieve the removal of closely related deer, even if the temporal spacing between culls is larger than a month. This is a result of surviving deer staying or returning to the area where a related individual was culled, which is encouraging as it suggests that culling is unlikely to be disrupting home ranges of surviving individuals to the point of promoting dispersal to new areas. Wildlife managers faced with the decision of (a) conducting culling in multiple locations in/surrounding first-priority areas for a shorter period of time or (b) prioritizing culling in first-priority areas for a longer period, might favor the latter to increase the chances of removing entire social groups of deer presumed to be infected with CWD. It would be interesting to analyze a temporal sequence to assess the number of days beyond which the probability of removing related individuals in a first-priority area is negligible.

## Figures and Tables

**Table 1:** Inferred full sibling (FS) and mother–offspring (MO) dyads in our samples of white-tailed deer (*Odocoileus virginianus*) culled in Minnesota, USA in 2019 and 2021. N: number of dyads; ♀: female; ♂: male. Geographic (Euclidean) distance between culling locations for members of the dyad and the number of days between culling of each individual are also provided.

	N	Distance (m)	Days Between Culls
**FS 2019**			
Fawn–fawn	12	<10	0–24
Fawn–fawn	1	376	15
Fawn–yearling (♂)	2	<10	0
**MO 2019**			
Adult (♀)–fawn	36	<10	0–46
Adult (♀)–fawn	11	20–1000	0–59
Adult (♀)–fawn	9	1000–11,217	1–48
Yearling (♀)–fawn	1	<10	0
Adult (♀)–yearling (♂)	3	<10	0–5
Adult (♀)–yearling (♂)	3	267–2573	6–37
Adult (♀)–yearling (♀)	6	<10	0–32
Adult (♀)–yearling (♀)	4	312–3779	2–41
**FS 2021**			
Fawn–fawn	5	<10	0–14
Fawn–yearling (♂)	1	<10	6
Fawn–yearling (♂)	1	50,489	10
Fawn–yearling (♀)	2	<10	0–27
Fawn–yearling (♀)	2	31–50,347	1–2
**MO 2021**			
Adult (♀)–fawn	32	<10	0–15
Adult (♀)–fawn	15	10–1000	0–52
Adult (♀)–fawn	4	1000–144,227	12–28
Yearling (♀)–fawn	1	20	19
Adult (♀)–yearling (♂)	3	0–586	1–8
Adult (♀)–yearling (♀)	6	<10	0–19
Adult (♀)–yearling (♀)	4	10–406	1–20
Adult (♀)–yearling (♀)	2	1148–136,592	1–4

## Data Availability

Data are permanently available at: Fameli, A. F.; Edson, J.; Walter, W. D. (2024). White-tailed deer from culling efforts in Minnesota. U.S. Geological Survey data release, https://doi.org/10.5066/P13BGPFC.
